# The Use of the Bath in Medicine

**Published:** 1913-01-25

**Authors:** 


### NEW DEVELOPMENTS IN MEDICINE.

### The Use of the Bath in Medicine.

Probably in few other departments of medicine has there been greater and more astonishing progress during the last decade than in that which is known as balneo-therapeutics—that is, treatment by bathing methods. Formerly the advantage of the "water cure" was well known, and in the early days of the last century there was a marked development of this side of treatment which resulted in the production of many bathing establishments, and the sudden popularity of various spas. But institutional bathing treatment in the general hospitals has, in this country at least, never been very much resorted to. It is true the method was used in cases of hyperpyrexia, and the wet pack, the cold and tepid sponge bath, and the dry pack were legitimate methods of treatment to be employed in various well defined ailments. But no hospital made a point of establishing a special department in which the method could be systematically used, and even today many of our largest institutions do not possess such a department.

Abroad they have moved much more rapidly. The balneo-therapeutic department is now a well recognised unit of every modern hospital on the Continent. It has attained its highest development in Germany, where the methods have always been favoured, and where their exponents have made a reputation for themselves by the skill and success with which they have applied the treatment. Berlin possesses one of the finest of these establishments in the shape of the University Hydro-therapeutic Institute, now surpassed by the fine bathing departments in connection with the Munich, Leipzig, and Hamburg hospitals. In America, too, the department is rapidly coming into its own, while similar progress has been made in Russia and Austria. Only in England and in France do the hospitals lag behind the times in this respect. In London, for instance, the only hospital that can lay claim to possessing a properly organised department of balneo-therapeutics is Charing Cross. To institutional workers it is of interest to note what is done in these departments. No one can justly urge that a hospital committee should sanction the expense necessary to establish and equip a new department for experimental purposes. Even the most up-to-date hospital must pause and consider whether new expense is justifiable. It must closely scrutinise what has been done in connection with the new apparatus it is asked to purchase, and it may have to consider whether similar good results are not possible without the use of such new apparatus and consequently with less expense and trouble. The question that hospital authorities have to decide, therefore, is this: Has the balneo-therapeutic department justified its existence? If so, at what cost and with what benefit to the institutions concerned?

This is a question that can be easily answered. Indeed, the best answer to it is the fact that no new hospital is planned in Germany without provision being made for such a department, either on an elaborate or primitive scale. Balneo-therapeutics is now a recognised aid to the physician and surgeon in treating hospital patients. It gives invaluable assistance in the treatment of such chronic conditions as arthritis and heart disease; it is successfully employed on nervous diseases, in affections of the digestive tract, in kidney trouble, and in certain acute conditions. Not the least valuable is its help in the treatment of obstinate skin diseases which have hitherto been the despair of hospital physicians. Balneo-therapy is, therefore, a help and has come to stay. What about its cost? Undoubtedly it is true that the initial expense of establishing and equipping a bathing department is very great, so great that it may be taken that in a hospital of two hundred beds a properly equipped department will raise the cost of the institution by as much as ten pounds per bed. But the experience of the German hospitals proves that it is a good investment. The cost of patients treated in the department compares more than favourably with the cost of those treated in, say, the genito-urinary department; where a large number of patients are treated and the cost is more evenly distributed the average expense is remarkably low. Full estimates of the cost per patient in the various departments, and the total average cost per bed are furnished annually in Ziegler's Handbook of the thirty-two principal German institutions.

It must not be thought that the department is merely a bathing department in the sense that the baths are only water baths. On the contrary, the great initial outlay on these departments is occasioned not so much by the provision of water-cooling and heating apparatus, but by providing electrical apparatus, and perhaps even more—owing to the amount of space required—for what is called sun-treatment. Sun baths are now rapidly coming into favour in the treatment of tuberculosis, and although there is a certain school which holds that such baths have little value unless they are given at a high altitude, the general opinion is that a sun bath, even at a low altitude, is a very helpful adjunct in the treatment of a phthisical patient. In a properly equipped bathing department in a German hospital—we may instance the Munich-Swabing Hospital—one finds some thirty kinds of baths, and almost as many methods of what are called manipulative treatment. Here, as elsewhere, the bathing department is combined with the massage department; the two go together and are intimately associated.

The cost of such an establishment is undoubtedly high, perhaps higher than it should be, but a great deal of useful work is being done in older hospitals which do not possess quite so elaborate an equipment. The success of the treatment—and there can be little question that it is successful and appreciated by patients and staff alike—is due largely to the care which is exercised in the selection of suitable cases and in the thorough manner in which the various methods are carried out. In the circumstances it may be asked, Are our hospitals justified in neglecting methods of treatment which have shown themselves so advantageous? We do not think they are. A modern hospital, to be truly modern, should neglect no opportunity to profit by the experience of its competitors. It should adopt what has been proved to be useful. And the balneo-therapeutic department has long since passed the experimental stage; it is now, as we have already affirmed, a well recognised part of the modern hospital. As such it fills a want which is very apparent in our own hospitals where the need for systematic treatment by baths is admitted. The question, in our opinion, is not principally one of expense; it is mainly one of ignorance. Our hospital staffs and committees have not yet realised what the benefits of such a department may mean to them. It is only in recent years that they have come to realise the necessity for special departments at all, and even with regard to these they do not follow their premises to a logical conclusion. We may illustrate this by citing the example of a London hospital. This institution some time ago decided that certain special departments should be established and placed in charge of specialists—a much needed and highly creditable reform. But the special departments have all been allocated to the surgical side, the medical staff apparently holding the view that while it was impossible for a general surgeon to specialise in anything and yet control a general ward it is not impossible for his colleague physician to run a special ward and a general ward at the same time. This illogical position will doubtless be reconsidered after a few years, but it serves to show that hospital staffs need waking up as much as do hospital committees. There is a certain kind of antagonism to departmental innovations which shows itself in ignorant denials of their utility. Against such antagonism it must be the duty of the enlightened committeeman to struggle and, if need be, to protest. Let him ask the first member of the medical staff he meets, "Why do we not have a balneo-therapeutic department?" and let him insist on an answer. Probably, we apprehend, the reply will be, "Because we have no money," in which case let him do his utmost to prevent a repetition of the reply by urging the needs of the institution upon the subscribers.

